# Posterior open-door laminoplasty secured with titanium miniplates vs anchors: a comparative study of clinical efficacy and cervical sagittal balance

**DOI:** 10.1186/s13018-019-1454-9

**Published:** 2019-11-28

**Authors:** Dongyue Li, Yong Hai, Xianglong Meng, Jincai Yang, Peng Yin

**Affiliations:** Orthopaedic Department, Chaoyang Hospital Affiliated to Capital Medical University, Beijing, 100020 China

**Keywords:** Posterior open-door laminoplasty, Titanium miniplates, Anchors, Clinical efficacy, Cervical sagittal balance

## Abstract

**Objective:**

Posterior open-door laminoplasty (PODL) is a common procedure for treating multilevel cervical spondylotic myelopathy (MCSM). Little information is available regarding the cervical sagittal balance and surgical efficacy of PODL when securing with different methods. Therefore, this study aims to investigate the clinical outcomes and the changes in cervical sagittal parameters and balance associated with PODL secured with titanium miniplates vs anchors.

**Method:**

A retrospective analysis was performed on the clinical data of 79 patients with MCSM who were treated in our institution from January 2015 to December 2016. Among them, 42 patients were treated by PODL secured with titanium miniplates (group A) and 37 patients by PODL secured with anchors (group B). Surgical time, intraoperative blood loss, hospital stay, hospitalized cost, VAS scores of neck pain, JOA scores, neck disability index (NDI), and improvement rate of spinal neurological function (IRNF) were recorded before surgery and at 12 months after surgery. Before surgery, at 1 month and 2 years after surgery, the following radiological parameters were recorded and compared on the lateral cervical X-ray images: the distance from the vertical axis of C2 sagittal plane to the posterior superior edge of C7 (C2-7 SVA), the inclusion angle of tangent between C2 and C7 trailing edge (C2-7 Cobb angle), and the intersection angle between the upper edge of T1 and the horizontal line (T1 Slope).

**Result:**

Comparing the two groups, there were no significant differences in surgical time, intraoperative blood loss, hospital stay, VAS, JOA, and NDI scores before surgery (*P* > 0.05); however, the hospitalized cost of group A were much higher than those of the group B (*P* < 0.05). At 2 years after surgery in the two groups, there was a significant reduction in VAS and NDI scores (*P* < 0.05), and JOA scores increased significantly (*P* < 0.05). In addition, there were no significant differences in VAS, JOA and IRNF between the two groups (*P* > 0.05); however, NDI scores of group A were better than those of group B (*P* < 0.05). In radiological parameters, before surgery, the two groups showed no significant differences in C2-7 SVA, C2-7 Cobb angle, and T1 slope (*P* > 0.05); however, after surgery, C2-7 SVA and T1 slope increased (*P* < 0.05), while C2-7 Cobb angle decreased (*P* < 0.05). At 2 years after surgery, the two groups did not differ significantly in C2-7 Cobb angle and T1 slope (*P >* 0.05), while C2-7 SVA of group A was superior to that of group B (*P* < 0.05). The difference value of C2-7 SVA measured before and after surgery was correlated negatively with that of NDI scores (*P* < 0.05).

**Conclusion:**

PODL secured with titanium miniplates or anchors achieved good clinical efficacy in the treatment of MCSM. However, the patients with miniplates feel a better cervical functional status, while those with anchors spend less on hospitalization. Both methods lead to anteversion of cervical spine, but cervical sagittal balance after miniplates is better than that of anchors.

## Introduction

Cervical degenerative disease is a major cause of spinal diseases, and its incidence has been rising in the elderly people [1–3]. Posterior open-door laminoplasty (PODL) is the primary treatment for multi-segmental cervical spondylotic myelopathy (MCSM), developmental cervical spinal canal stenosis, ossification of cervical posterior longitudinal ligament, and so on. Studies have shown this surgical approach has definite and enduring efficacy in releasing spinal compression and improving the function of nervous system [1–7]. After PODL, it is usually necessary to firmly fix the opened laminae in order to maintain the enlargement and decompression of the spinal canal. To do so, the commonly used methods include silk suspension, suture anchor, and titanium miniplate (centerpiece), which can pry open the lamina on the open-door side [1, 2, 5–10].

Silk suspension is to tie the silk into the space between the spinous process and articular capsule of the door-shaft side. But this method has lower biomechanical strength, and re-closure of the opened lamina may occur, resulting in relapse of the neurological symptoms [1, 8]. It has been reported that PODL secured with either miniplates or anchors can achieve a good clinical effect [1, 2, 8, 10], but few reports are concerned with the comparison of the two approaches. Whatever the approach, damage to muscle group and ligaments of the neck seems inevitable, which may further disrupt cervical sagittal balance, leading to straightening of normal cervical lordosis or even kyphosis [11–13]. More studies are devoted to coronal-sagittal balance of the lumbar spine and the influence of spinal-pelvic parameters on postoperative lumbar spine [14, 15], while few are focused on the postoperative cervical sagittal parameters and changes in cervical sagittal balance.

In this study, clinical efficacy and radiologic parameters were compared between PODL secured with titanium miniplates and anchors. The influence of different surgical approaches on cervical sagittal balance was discussed, and the potential correlation between cervical sagittal parameters and clinical efficacy was analyzed.

## Methods

### Clinical data

From January 2015 to December 2017, 79 patients with MCSM were recruited in our institution. They were selected according to the following criteria. Inclusion criteria include the following: Sensory and motor disorders of the four limbs or sphincter of Oddi dysfunction, confirmed as MCSM by cervical X-ray, CT, MRI and neurological examination; compressed cervical segments ≥ 3 by preoperative MRI; these patients received PODL in the C3-7 vertebrae; they were followed up for at least 2 years after surgery, and all of them had radiologic data; all of them received bone density examination before surgery, and none of them had severe osteoporosis, with T value> − 2.5. Exclusion criteria were as follows: Non-degenerative cervical diseases such as trauma, deformity, tuberculosis, tumor, and so on.

Before surgery, all patients were informed of the advantages and disadvantages of the two surgical approaches without any bias, and they were allowed to make their own choice. Depending on the surgical approach, the patients were divided into two groups: (1) PODL secured with titanium miniplates (centerpiece) (group A) and (2) PODL secured with anchors (group B). All surgeries were performed by the same group of surgeons.

### Surgical procedures

After general anesthesia, a Mayfield head clamp was used to immobilize the head and neck in a flexed position under the prone position, with the trunk slightly elevated. A posterior midline approach was adopted. The skin, subcutaneous tissues, and nuchal ligament were cut open layer by layer. The laminae from C3 to C7 were exposed by stripping the paravertebral muscle from the spinous process and laminae to the medial margin of bilateral facet joints. The more severely affected side was taken as the open side, and the contralateral side as the door-shaft side. If the symptoms were of comparable severity, then the left side was taken as the open-door side, and the right side as the door-draft side. A groove was made at the boundary between bilateral facet joints and laminae. The entire layer of lamina was severed on the open-door side, and the inner layer of cortex was preserved on the door-shaft side. The ligamentum flavum were severed at C2-3 and C7-T1 on the open-door side, and the laminae of each segment were opened successively.

*Group A (Titanium miniplates)*: Centerpiece miniplates of appropriate length was installed between the lateral mass and opened lamina; the opened lamina was held with the claw-shaped clamp and immobilized with one to two titanium pins; another two titanium pins were used to immobilize the lateral mass on the open-door side.

*Group B (Anchors)*: Five silk anchors were inserted into the lateral mass on the door-shaft side. A hole was drilled on the opened spinous process, with the silk passing through the hole to immobilize the opened laminae. Strict hemostatic measures were adopted, and the incision was washed with normal saline. A drainage tube was dwelled outside of the spinal dura mater, and the incision was sutured layer by layer.

Antibiotics were given prophylactically for up to 48 h after surgery. Considering larger surgical incisions and more drainage volume, the drain was kept for more than 48 h in all patients. When the drainage volume was less than 50 ml/24 h, the drain would be removed. The patients were assisted in off-bed movement wearing cervical collar. They began to take exercises of posterior cervical muscles after 3 or 4 weeks.

### Efficacy evaluation and radiographic assessment

Surgical time, intraoperative blood loss, hospital stay, hospitalized cost, and Visual analogue score (VAS) of neck pain were recorded in the two groups. Before surgery and at 2 years after surgery, JOA [16] (Japanese Orthopaedic Association) scale ranging from 0 to 17 points was used to assess the spinal functions. Improvement rate of spinal neurological function (IRNF) was calculated as follows: [(Postoperative JOA score − preoperative JOA score)/(17 − preoperative JOA score) × 100%]. IRNF was assessed postoperatively for nerve function on this basis. Neck disability index (NDI) [17] was used to assess the cervical functional status. NDI ranged from 0 to 50, and the lower the score, the lower the severity of cervical dysfunction.

**Fig. 1:**
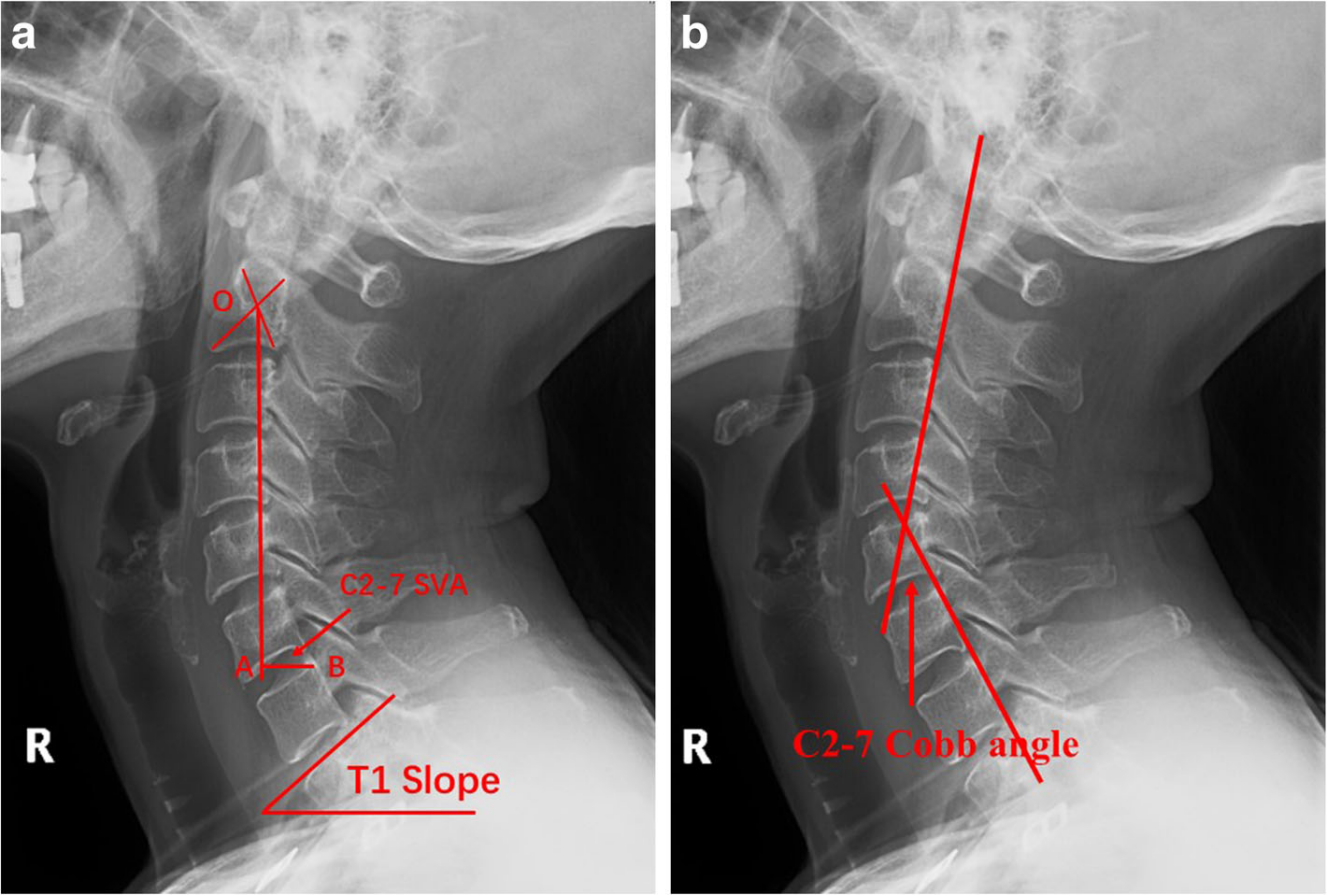
Measurement of cervical sagittal imaging parameters. **a** C2-7 SVA is the distance between the vertical axis of C2 sagittal plane (OA) and the posterior superior edge of C7 (point B), that is, the length of AB. T1 slope is the intersection angle between the upper edge of T1 and the horizontal line. **b** C2-7 Cobb angle is the inclusion angle of tangent between C2 and C7 trailing edge

All patients received standard lateral cervical X-ray before surgery and at 1 month and 2 years after surgery. It was required that the superior margin of T1 vertebra was exposed. The radiologic parameters were measured by one orthopedist and one radiologist. The means were taken of all measurements. Parameters of cervical sagittal balance measured and recorded from the standard lateral cervical X-ray images at the above-mentioned three time points included the following: the distance between the vertical axis of C2 sagittal plane and the posterior superior edge of C7 (C2-7 sagittal vertical axis; SVA), the inclusion angle of tangent between C2 and C7 trailing edge (C2-7 Cobb angle), and the intersection angle between the upper edge of T1 and the horizontal line (T1 slope) (Fig. 1).

**Fig. 2:**
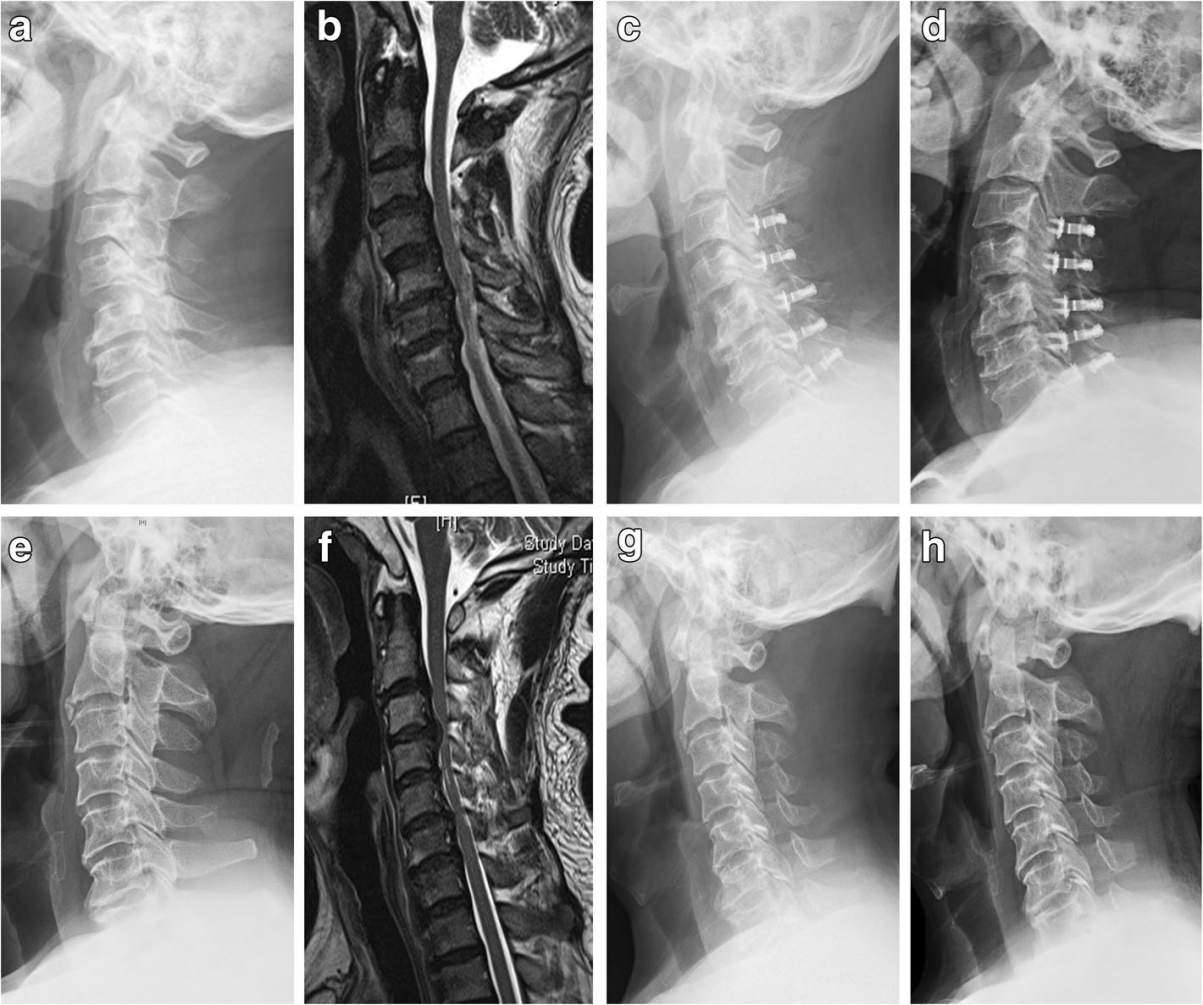
Comparison of cervical sagittal imaging parameters between two surgical approaches. **a**–**d** PODL secured with titanium miniplates (centerpiece). **a** Preoperative lateral cervical X-ray showed cervical degenerative and multi-segment spinal stenosis, with C2-7 SVA of 10.6 mm. **b** Preoperative cervical MRI showed multi-segment spinal compression at C3-7 segments. **c** At 1 month after surgery, lateral cervical X-ray showed that there was apparent increase of cervical canal width, with significant cervical anteversion and C2-7 SVA of 29.6 mm. **d** At 2 years after surgery, lateral cervical X-ray indicated good position of the titanium miniplates, and there was significant improvement of cervical anteversion, with the C2-7 SVA of 12.1 mm. **e**–**h** PODL secured with silk anchors. **e** Preoperative lateral cervical X-ray showed cervical degenerative and multi-segment spinal stenosis, with C2-7 SVA of 20.5 mm. **f** Preoperative cervical MRI showed multi-segment spinal compression at C3-7 segments. **g** At 1 month after surgery, lateral cervical X-ray showed that there was apparent increase of cervical canal width, with significant cervical anteversion and C2-7 SVA of 33.6 mm. **h** At 2 years after surgery, lateral cervical X-ray indicated good position of the anchors, and there was mild improvement of anteversion, with the C2-7 SVA of 29.1 mm

### Statistics analyses

Statistical analyses were performed using SPSS 17.0 software. Measurements were expressed as ($\overline{x}$ ± SD). Paired-samples *t* test was performed for intragroup comparison, and independent *t* test for intergroup comparison. Several measurements of the same radiologic parameters were compared by using repeated measures analysis of variance (ANOVA). The relationship between radiologic parameters and clinical indicators was tested by Pearson’s correlation analysis. *P* < 0.05 indicated significant difference.

## Results

### Surgical results

Group A included 42 patients, including 27 males and 15 females with an average age of 61.2 ± 8.8 years old (47–77 years old) and average course of 45.3 ± 7.7 months; group B included 37 patients, including 23 males and 14 females with an average age of 60.9 ± 8.3 years old (45–76 years old) and average course of 43.7 ± 9.4 months. All patients were followed up for at least 2 years. The average time of drain removal was 3.10 ± 0.94 days. In group A, one patient developed epidural hematoma after operation, and the clinical symptoms of the patient improved significantly after timely removal of the hematoma. Group B had one case of wound infection. After debridement and anti-infection treatment, the wound healed well. Other patients did not have such complications as neurovascular injury, cerebrospinal fluid leakage, loosening of internal fixation, and so on.

### Clinical results

Comparing the two groups, there were no significant differences in surgical time, intraoperative blood loss, hospital stay, VAS, JOA, and NDI scores before surgery (*P* > 0.05); however, the hospitalized cost of group A were much higher than those of the group B (*P* < 0.05). At 2 years after surgery, there was a significant reduction in VAS scores of neck pain (group A *t* = 8.573, group B *t* = 4.743, *P* < 0.05); JOA scores increased significantly (group A *t* = 15.490, group B *t* = 24.652, *P* < 0.05); NDI scores decreased significantly (group A *t* = 26.119, group B *t* = 17.683, *P* < 0.05). At 2 years after surgery, neither were there significant differences in JOA scores and IRNF between the two groups (*P* > 0.05); however, NDI scores of group A were better than that of group B (*P* < 0.05) (Table 1).

**Table 1:** Baseline information, VAS, JOA, and NDI scores in the two groups ($\overline{x}$ ± SD)

Item	Group A (*n* = 42)	Group B (*n* = 37)	*t* value	*P* value
Age (years)	61.2 ± 8.8	60.9 ± 8.3	0.110	0.913
Course of disease (months)	45.3 ± 7.7	43.7 ± 9.4	0.580	0.565
Surgical time (min)	116.1 ± 16.7	113.9 ± 15.1	0.782	0.439
Intraoperative blood loss (ml)	184.4 ± 35.8	170.4 ± 34.3	1.272	0.211
Hospital stay (days)	7.6	7.5	0.525	0.603
Hospitalized cost (Yuan)	104112.3	67331.5	18.768	0.000
VAS scores of neck pain				
Before surgery	2.7 ± 0.92	2.5 ± 1.04	0.961	0.342
At 2 years after surgery	1.3 ± 0.65	1.4 ± 0.50	0.541	0.592
JOA scores				
Before surgery	8.8 ± 1.09	8.4 ± 0.97	1.304	0.200
At 2 years after surgery	13.5 ± 1.29	12.9 ± 1.37	1.247	0.230
IRNF	56.9 ± 13.5	49.6 ± 12.9	1.790	0.081
NDI scores				
Before surgery	42.7 ± 3.04	42.3 ± 2.76	0.280	0.718
At 2 years after surgery	22.2 ± 2.44	24.4 ± 3.62	2.501	0.017

### Radiographic results

#### Cervical sagittal parameters on X-ray

Measurements of radiologic parameters in the two groups are shown in Table 2. Within each group, after surgery, both C2-7 SVA and T1 slope increased significantly (*P* < 0.05), while the C2-7 Cobb angle decreased (*P* < 0.05). There were significant changes in C2-7 SVA, C2-7 Cobb angle, and T1 slope on sagittal parameters at 1 month and 2 years after surgery in each group (group A *t* = 4.051, *t* = 13.180, *t* = 7.795, *P* < 0.05; group B *t* = 14.205, *t* = 15.848, *t* = 3.321, *P* < 0.05).

**Table 2:** Cervical sagittal parameters on X-ray in the two groups ($\overline{x}$ ± SD)

Item	Group A (*n* = 42)	Group B (*n* = 37)	*t* value	*P* value
C2-7 SVA (mm)				
Before surgery	20.3 ± 6.9	20.1 ± 5.4	0.856	0.394
At 1 month after surgery	34.8 ± 5.7	33.9 ± 5.1	1.767	0.085
At 2 years after surgery	22.4 ± 4.1	24.9 ± 4.7	3.113	0.002
F value ^*^	6.978	161.014		
*P* value*	0.006	0.000		
C2-7 Cobb angle (°)				
Before surgery	21.4 ± 6.6	21.1 ± 5.8	0.834	0.406
At 1 month after surgery	14.8 ± 3.8	14.2 ± 3.3	0.799	0.426
At 2 years after surgery	19.5 ± 6.3	18.5 ± 5.1	1.693	0.093
F value ^*^	124.228	49.836		
*P* value*	0.000	0.001		
T1 Slope (°)				
Before surgery	24.7 ± 2.4	24.4 ± 3.8	1.025	0.307
At 1 month after surgery	33.2 ± 3.5	32.9 ± 3.2	1.166	0.246
At 2 years after surgery	26.5 ± 2.7	28.1 ± 3.1	1.814	0.072
F value ^*^	26.133	33.135		
*P* value*	0.001	0.000		

Note: *indicates statistics of intragroup comparison by using repeated measures ANOVA

The sagittal parameters of C2-7 SVA, C2-7 Cobb angle, and T1 slope were not significant differences (*P >* 0.05) between the two groups before surgery and at 1 month after surgery. At 2 years after surgery, no significant differences were found in C2-7 Cobb angle and T1 slope (*P* > 0.05); however, there were significant differences in C2-7 SVA (*P* < 0.05), and group A was superior to that of group B. (Table 2). For description of representative cases, see Fig. 2.

#### Relationship between radiologic parameters and clinical indicators

According to statistical analyses, when comparing the two groups at 2 years after surgery, there were significant differences in NDI scores and C2-7 SVA (*P* < 0.05). To facilitate statistical comparison, the difference value in NDI scores and C2-7 SVA at 2 years after surgery between the two groups were calculated (Table 3). Pearson correlation coefficients were calculated. The difference value in NDI scores was correlated negatively with that in C2-7 SVA (*r* = − 0.433, *P* < 0.05).

**Table 3:** Correlation analysis between radiologic parameters and clinical indicators ($\overline{x}$ ± SD)

Item	Group A	Group B	Difference in NDI at 2 year after surgery	
			R value	*P* value
Difference in NDI	21.2 ± 2.9	17.9 ± 3.7		
Difference in C2-7 SVA (mm)	2.21 ± 0.71	4.77 ± 1.04	− 0.433	0.005

## Discussion

MCSM can cause severe damage to the spinal function. Given to the grim consequences of progression, early surgery is important after confirmed diagnosis, so as to release spinal compression [1–8]. When three or more segments are involved, posterior cervical approach is preferred to ensure the clinical outcomes and safety [1, 2, 6]. PODL is one of the common treatments for MCSM, which can increase vertebral canal volume and release compression of spinal cord and nerve roots, and the aim is to create enough room for the recovery of the nerves and spinal cord [1, 2, 6, 7].

In our study, PODL was performed using either titanium miniplates or anchors. Silk is fixed in screw, and they are made into a unity of anchor. The unity can prevent slip and rupture of the silk at the root of the screw in the lateral mass; moreover, the anchor has locking function and can prevent screw dislocation, which may otherwise lead to reclosure. This method proves to have a reliable open-door effect. Titanium miniplate [1, 2, 7, 8] is to install the centerpiece miniplate between the lamina and lateral mass on the open-door side, so as to enlarge the vertebral canal and to release spinal compression. The rigid support provided by the centerpiece miniplate can effectively prevent postoperative reclosure or loss of door-closing amplitude, so as to ensure the clinical efficacy. In our study, group A received immobilization with titanium miniplates, and group B immobilization with silk anchors. At 2 years after surgery, VAS, JOA, and NDI scores were all improved significantly than before (*P* < 0.05). This indicated that either approach could achieve a satisfactory clinical effect. It was found that at 2 years after surgery, NDI scores of group A were much better than those of group B (*P*<0.05). NDI is a subjective evaluation of patients’ cervical functional status. Several items of NDI deal with the subjective perception of comfort. Though bias seems inevitable with NDI, postoperative cervical functional status of the patient does have a considerable impact on the patients’ life quality [18]. We think that NDI has a certain clinical significance and serves as an important measure for surgical outcomes. Although PODL-secured titanium miniplates incurred greater hospitalized cost than using anchors (*P* < 0.05), the former provided higher cervical comfort for the patient after surgery.

Cervical sagittal balance is an important component of balance of the entire spine. Changes in cervical sagittal parameters after PODL will affect sagittal balance of the cervical spine or even the entire spine [11–13]. C2-7 SVA, C2-7 Cobb angle, and T1 slope are important measures of cervical sagittal balance [11–13, 19]. After surgery, both C2-7 SVA and T1 slope increased in the two groups than before (*P* < 0.05), while C2-7 Cobb angle decreased (*P* < 0.05). The result indicated conspicuous postoperative cervical anteversion. One major cause might be the damage to posterior cervical muscle-ligament complex when exposing the bilateral lamina. Additional cause might be that during door-opening operations in C3 and C7, attachments of the muscle in the spinous processes of C3 and C7 might be damaged with severing of laminae from the lateral mass on the open-door side. The above factors may lead to damage of muscle group and bony structure, which further affects *cervical instability*. As a result, anteversion may occur due to failure to maintain normal cervical curvature. Cervical anteversion is most significant at an early stage after surgery. Along with the progression of time, many patients may still fail to recover to preoperative curvature. Disruption of cervical sagittal balance as measured by C2-7 SVA, C2-7 Cobb angle, and T1 slope at 1 month after surgery was most apparent. These parameters were improved (*P* < 0.05) at 2 year after surgery, but cervical sagittal balance still did not recover to the preoperative curvature (*P* < 0.05).

However, controversy is still going on as to which radiologic parameter is of the highest clinical significance among all parameters of cervical sagittal balance. According to Ling et al. [19], C2-7 SVA was the most important radiologic parameter among the followings: C0-2 Cobb angle, C2-7 Cobb angle, C4-7 Cobb angle, C0-7 SVA, C1-7 SVA, C2-7 SVA, C7 Slope, T1 slope, angle of cervical endplate, McGregor's slope, elbow angle, craniospinal angle, and thoracic inlet angle. In this study, both groups had a significant increase in C2-C7 SVA after surgery (*P* < 0.05), indicating cervical anteversion. But at 2 years after surgery, group A achieved a much better recovery than group B (*P* < 0.05). Immobilization with silk anchors might cause complete damage to the bony structure on the open-door side. As a result, the cervical spine loses the support offered by bony structure on this side, leading to cervical instability to varying degrees. Moreover, posterior cervical muscle group was damaged, and the normal physiological curvature of the cervical spine could not be effectively maintained, leading to persistent anteversion [20]. However, miniplates provides a stable bridging fixation between the opened lamina and ipsilateral lateral mass, so that the opened lamina and lateral mass for the same segment can form a whole unity. In the meantime, rigid fixation can be provided for the open-door side, so that the original support by the body structure can be restored as much as possible. The opened lamina will be subjected to uniform stress under flexion and extension and rotation of the cervical spine, thereby increasing the possibility of restoring the normal cervical stress [21]. Besides, immobilization with miniplates can also provide solid stabilization to the door-shaft side, which is conducive to bony union on this side [7]. Cervical anteversion after PODL secured with miniplates might be only the result of damage to the posterior muscle group.

As to whether the radiologic parameters of cervical sagittal balance would affect clinical symptoms of the patients, Tang et al. [11] reported 113 patients receiving PODL with multilevel fusion. It was found that C2-7 SVA was correlated negatively with SF-36 and cervical anteversion would affect the surgical efficacy. Under normal physiological curvature of the cervical spine, posterior muscle group has the lowest energy consumption, and therefore, the patient has the highest degree of comfort. But if cervical anteversion occurs due to damage of bony structure and posterior muscle group after PODL, the posterior muscle group no longer has the lowest energy consumption [19]. This will further lead to such clinical symptoms as fatigue, discomfort, and pain of the posterior neck. Two different approaches were adopted in this study. The results showed that the difference value in NDI scores was correlated negatively with that in C2-7 SVA (*r* = − 0.433, *P* < 0.05). This means disruption of cervical sagittal balance would definitely impair cervical functional status, which further lowered cervical comfort of patients.

However, the present study still had the following limitations: (1) The sample size was small. (2) The duration of follow-up was limited. Our conclusions need to be further verified through studies with larger sample size and longer follow-up.

## Conclusion

Taken together, PODL secured with titanium miniplates or anchors could achieve satisfactory improvement of neurological function for MCSM. However, the use of miniplates led to better cervical functional status, while the use of anchors reduced the hospitalized cost. Significant changes occurred in cervical sagittal balance after surgery using either approach, mainly presenting as cervical anteversion, which was most serious at the early stage after surgery. But this condition would improve with time, and alteration of sagittal balance had an impact on cervical comfort. It was revealed that the recovery of cervical sagittal balance was better secured with the miniplates than anchors.

## Data Availability

All data used and analyzed during this study are available from the corresponding author upon reasonable request.

## Abbreviations

ANOVA: Analysis of Variance
IRNF: Improvement rate of spinal neurological function
JOA: Japanese Orthopaedic Association
MCSM: Multi-segmental cervical spondylotic myelopathy
NDI: Neck disability index
PODL: Posterior open-door laminoplasty
SVA: Sagittal vertical axis
VAS: Visual analogue score
